# Use of social networks, video games and violent behaviour in adolescence among secondary school students in the Basque Country

**DOI:** 10.1186/s40359-022-00947-w

**Published:** 2022-10-27

**Authors:** Álvaro Moro, Marta Ruiz-Narezo, Janire Fonseca

**Affiliations:** 1 Deusto Institute of Drug Dependencies, University of Deusto, Bilbao, Spain; 2 Faculty of Education and Sports, University of Deusto, Bilbao, Spain

**Keywords:** Adolescence, Social network, Video games, Violent behaviour

## Abstract

This article is aimed at exploring the relationship between abusive use of the internet and social media and involvement in violent behavior in adolescence. This analysis used a sample of 2549 participants of students enrolled in secondary education in the Basque Country, including lower secondary education, baccalaureate studies and vocational training courses. The results of this study show that the antisocial behaviour and bullying variables are associated with the different categories of use of information and communication technology. Gender differences appear to be irrelevant in this correlation as it is similar in both boys and girls. These data must be taken into consideration by the educational community, which has been shown to have a protective influence and by school guidance departments in particular, which are designed specifically to tackle these kinds of situations.

## Introduction

Adolescence is the transition period from childhood to adulthood that includes some major changes. It refers to a complex biopsychosocial maturing process involving variables that operate independently from one another: these include biological variables (physiological changes as puberty occurs and the brain matures), psychological variables and social variables [1]. In other words, adolescence may be defined as the process by which individuals attain autonomy, responsibility and psychological and social adulthood [2]. Adolescents may face difficulties at the individual, social/community, school, peer group and family level as they mature [3, 4]. Adolescent risk behaviours are common and are associated with the experimentation inherent to this life stage, which is a highly complex period [5, 6]. Although the social alarm that is regularly aroused by these risk behaviours is often irrelevant as they disappear as quickly as they emerged [7], we believe it is important to explore the interaction between three behaviours that are becoming more and more prevalent: antisocial behaviour, bullying [8] and abuse of different kinds of technology [9–12].

More specifically, our interest lies in studying the relationship between abusive use of the internet [13] and social media and involvement in antisocial behaviour and bullying on the premise that the levels of violence observed in the online world reinforce aggressive, violent behaviour in the real world [14]. Scholars such as Andrews [15] show the existence of profiles based on social indicators that are directly linked to young people’s positioning in the online world, which is vital in understanding patterns of interaction and behaviour among young people.

Bullying at school and involvement in violent or antisocial behaviours are considered adolescent risk behaviours. Involvement in these behaviours may be caused by multiple risk factors associated with the adolescent themselves and the microsystems surrounding them [5]. In this article, we explore involvement in social media (internet) and video games, their effect on adolescents’ behaviour and the association between violent behaviour and (ab)use of the internet.

Problems associated with abusive internet use and prioritisation of the digital world by adolescents are on the rise, with many adopting a new lifestyle centred around the internet [13] as they find it increasingly difficult to separate the online and offline worlds [16]. It is important to study this emerging issue, which is made all the more complex by its association with other adolescent risk behaviours.

Research in this area should take an educational approach and include all stakeholders to ensure that a holistic, comprehensive response to the problem can be developed. We will now describe the behaviours that will be analysed in this study in greater detail.

### Adolescent risk behaviours

#### Abusive use of technology

The use of new technology (ICT) has risen exponentially throughout the last decade in particular. Technologies designed for communication and obtaining information, such as the internet and mobile phones, have become especially popular [17, 18]. Due to their multiple benefits and advantages, these technologies have become an integral part of our everyday lives. These days, people use technology to communicate, socialise, look for a partner, express their feelings or buy products and services.

ICT helps to fulfil adolescents’ needs for autonomy, contributing to the search for new sensations and the establishment of affective bonds and relationships [19, 20]. Social media allows users to adopt an identity that would be unacceptable in the real world, so adolescents are able to create a character and be who they really want to be. In addition, the internet [13] offers adolescents a new way of meeting people, building their confidence and self-esteem as they become part of a group and obtain emotional support [21–23]. As a result, the internet has become a key site for socialisation and has heightened the need for constant interaction between adolescents and their peers [24, 25].

Several research studies, including Copeland et al. [26] agree that social media should be used as a beneficial resource but that their potential to become a source of risk should not be overlooked. Although the internet, social media and online games are not negative in themselves, they can become problematic when people use them to cope with everyday issues and stressors, such as loneliness and [27, 28] or when they are used to access inappropriate content, giving rise to physical, mental, psychological and social problems [29], as well as affecting socialisation, perceptions of sexual relationships, academic performance [30], etc. In this regard, Griffiths (cited in [31]), states that clinical criteria may be used to determine homogeneous alignment between chemical and behavioural addiction and presents the following parameters for measurement: prioritisation of an activity that dominates the individual’s thoughts, feelings and behaviours; mood changes; tolerance; withdrawal symptoms or discontent when levels of activity are reduced; intrapsychic conflict and potential for relapse.

These factors may be associated with involvement in other risk behaviours, especially violence. Studies have shown that involvement with computer or video games is related to aggressive behavior [32], and with reduce pro-social behavior [33]. In addition, the constant use of the Internet does influence the loss of control and therefore maintain an interference with the daily life of students [34] and it must be seen as a new risk factor in the research about school violence perpetration [35].

#### Antisocial behaviour and bullying

Antisocial behaviour is understood as a range of actions that cause harm to others, which frequently take the form of aggression, or that breach social norms and violate other people’s rights [36]. Specific behaviours are labelled as antisocial on the basis of a social judgement regarding the severity of the acts committed and their divergence from social norms in a particular society.

Classifications, typologies and definitions of bullying have been drawn up by a variety of scholars [37–40], who concur that aggression, intent to harm and recurrence are the main characteristics of bullying [41, 42], differentiating between physical bullying, verbal bullying and relational bullying [36, 37].

In 2015, the term ‘cyberbullying’ was added to thesauruses [39, 43], referring to the use of mobile devices and social media to bully a victim who is unable to defend themselves [44, 45] A number of studies have cast light on the consequences of cyberbullying, which often have a long-lasting effect on the victims [46], including antisocial behaviour [47] anxiety, sadness, helplessness, frustration, anger, stress, somatisation, isolation, substance abuse, internet addiction, absenteeism, poor academic performance, low self-esteem, sleep problems, depression, suicidal ideation, suicide attempts and suicide [24, 48].

Participation in violent or criminal acts is linked to social status in secondary schools, as several studies such as Andrews et al. [49] have shown. Domínguez and Portela [50] shows that violence is becoming increasingly widespread among young people on social media, and differences by gender have been observed in many research studies [51]. Boys are more frequently involved in aggressive behaviour, while girls are more likely to engage in victimizing behaviour [52, 53]. Both types of behaviours are associated with domination, discrimination and abuse of power, as highlighted by Ortega-Barón et al. [54]. The fact that bullies often remain anonymous in this context also has a considerable impact on the victims. Meanwhile, Muñiz et al. [55] argue that violence on social media is linked to gender and types of use: while girls tend to use the internet and social media for utilitarian purposes, boys tend to use them more for entertainment.

According to Cowie [56], victims of cyberbullying suffer similar psychological issues as victims of traditional bullying, including depression, high levels of social anxiety and low self-esteem, with a direct impact on their academic performance. Schenk and Fremouw [57] add that cyberbullying victims are also affected by frustration, stress, anger, difficulties concentrating and sadness, with a small proportion also experiencing suicidal ideation.

## Methodology^1^

### Sampling

For this exploratory analysis of the relationship between abusive use of the internet and social media and involvement in violent behaviour, data from the DROGAS Y ESCUELA IX. Encuesta sobre Uso de Drogas en Enseñanzas Secundarias en La CAPV [DRUGS AND SCHOOL IX: Survey on Drug Use at Secondary Schools in the Autonomous Community of the Basque Country] study was used. The survey covered a representative sample of students enrolled in secondary education in the Basque Country, including basic secondary education, baccalaureate studies and vocational training courses.

With regard to the sampling procedure, a two-stage cluster sampling was carried out, in which, in the first instance, educational centres (first-stage units) were randomly selected and in the second-place classrooms (second-stage units), providing the questionnaire to all the pupils present in the classrooms. The sample comes from a database of 6007 questionnaires from 43 educational centres. For this analysis, a sample of 2549 participants of the indicated educational stages was used for the antisocial behaviour scale and 2926 questionnaires for the school violence scale. With a school population of 140,000 students in secondary education in the Basque Country, the maximum sampling error for a confidence level of 95% and p = q = 0.5 are 2% and 1.8%respectively.

### Instrument

The data used for the analysis were taken from the DRUGS AND SCHOOL IX: Survey on Drug Use at Secondary Schools in the Autonomous Community of the Basque Country, which is a survey of students in secondary education carried out for the Basque Government by the Deusto Institute of Drug Dependency. The survey was conducted at secondary education facilities to ascertain the level of drug use among Basque adolescents and explore a series of associated variables.

#### Use of information and communication technology

To analyse the use of information and communication technology, the number of hours each weekday that students used video games, online games, internet, social media and mobile phones was recorded. Four different categories were created: No use; Moderate use—less than 2 h per day; Intensive use—2–4 h per day; Abusive use—more than 4 h per day. Although in the Drugs and School research a specific questionnaire on problematic Internet use (CERI) was applied [58] we preferred to talk about the use of information and communication technologies rather than problematic use. Our idea is to analyse the association of the number of hours spent (as an indicator) with the rest of the variables.

#### Antisocial behaviour

The instrument used for the study was an adaptation of the Escala de Conducta Antisocial y Delictiva en Adolescentes—ECADA [Antisocial and Criminal Behaviour in Adolescents Scale] [59]. The scale contains 20 items relating to different antisocial behaviours and adolescents are asked to indicate whether or not they have engaged in these behaviours in the last year and how frequently (1—Less than 5 times; 2–5—10 times; 3—more than 10 times). The total score for the scale is the sum of the values in each category and can range from 0 to 60 [60].

More than an adaptation, it has been an extension of the positive responses to the items in order to measure not only antisocial action but also its frequency in the last year. Thus, on the one hand, it has been possible to extend the range of responses from 0 to 60 in the total score of the scale and, on the other hand, in its interpretation, instead of placing a cut-off point to consider serious antisocial behaviour, an average of the participants’ score on this scale has been used.

The resulting mean was 3.77 with a bias-corrected and accelerated (95%) confidence interval (BCa) of 3.55–4.01. In addition, the psychometric properties of the scale were evaluated: a high internal consistency was observed for the antisocial behavior scale (alpha 0.910–omega 0.911) [60].

#### Bullying

Information on bullying has been collected through 4 items with four response categories to measure the frequency of physical, verbal, sexual violence and social exclusion. It features 4 items listing bullying behaviours and adolescents are asked to state whether or not they have engaged in these behaviours in the last year and how frequently (1—Never; 2—Once or twice; 3—3 or more times). The total score for the scale is the sum of the values in each category and can range from 0 to 8. The questionnaire has been used in previous editions of the Drugs and School series [61] and collects the three major modalities of bullying [62] the one exercised directly, the one exercised indirectly and as already done by the ISEI-IVEI [63] (2017) the modality of cyberbullying. In addition, the psychometric properties of the bullying scale were evaluated and a sufficient internal consistency was observed (alpha 0.637–omega 0.646) [59]. considering that it only consists of four items.

## Analysis and results

Firstly, a descriptive analysis of the aforementioned variables was carried out. As shown in Table 1, in a first observation of the descriptive results we can see that the mean scores of antisocial behavior and bullying increase as the frequency of use of video games, online games, Internet, social networks and cell phones increases in many of the categories of variables analysed.

**Table 1:** Mean scores for antisocial behaviour and bullying (total and by type of use)

	Ant. social behav.	Bull.	Ant. social behav.	Bull.	Ant. social behav.	Bull.	Ant. social behav.	Bull.
*No use*								
M	3.13	0.39	3.09	0.41	2.68	0.49	3.4	0.76
N	1037	1090	1348	1410	374	392	63	68
SD	5.336	0.941	5.09	0.925	5.879	1.173	5.604	1.271
*Moderate use*								
M	3.84	0.58	4.12	0.58	3.48	0.47	2.67	0.42
N	1091	1140	840	888	1115	1171	1005	1057
SD	6.293	1.079	6.902	1.05	6.387	0.929	5.291	0.922
*Intensive use*								
M	4.95	0.71	5.32	0.89	3.92	0.49	3.88	0.48
N	232	249	190	200	567	596	653	690
SD	8.167	1.166	7.954	1.456	5.677	1.005	6.321	0.955
*Abusive use*								
M	5.86	0.77	6.5	0.84	5.26	0.72	5.12	0.68
N	132	147	98	106	419	447	766	812
SD	9.852	1.298	11.141	1.487	7.383	1.259	7.537	1.246
*Total*								
M	3.75	0.52	3.75	0.52	3.76	0.52	3.76	0.53
N	2492	2626	2476	2604	2475	2606	2487	2627
SD	6.403	1.054	6.381	1.054	6.384	1.051	6.412	1.056
	Video games		Online games		Internet and social media		Mobile phones	

The distribution of the quartiles and the histogram were taken into consideration in analysing the distribution of the variables, as antisocial behaviour and bullying were suspected to be non-normal variables. To confirm this, a goodness-of-fit test was carried out to confirm the type of distribution of the data and the type of statistical test that should be carried out in the statistical contrast (parametric or non-parametric).

The goodness-of-fit test used was the Kolmogorov–Smirnov or K–S test, which is a test of statistical significance to check whether the data in a sample are normally distributed. It is used for continuous quantitative variables and for samples exceeding 50.

The Z-score was 0.279 for the antisocial behaviour variable and 0.386 for the bullying variable; in both cases, the statistical significance (bilateral asymptotic significance) or *p* value was 0.000. Since the *p* value was less than 0.05, H0 was rejected. In other words, the antisocial behaviour variable and the bullying variable do not follow a normal distribution, so non-parametric tests must be used for the statistical contrast [64, 65].

A comparative analysis was then carried out using the most common tests, the Mann–Whitney U test and the Kruskal–Wallis test, to check for significant differences in the distribution of different categories of variables or for differences between the groups. A 95% confidence level is used to test for statistically significant differences (0.05). This test was carried out on the overall study population and the sample was then segmented by gender to explore possible differences in the relationships between the variables among boys and girls.

With regard to antisocial behaviour, all the tests carried out (see Table 2) rejected H0 and found significant differences in antisocial behaviour in relation to the use of video games, online games, internet, social media and mobile phones.

**Table 2:** Contrasts for antisocial behaviour hypothesis (total sample and by gender)

Kruskal–Wallis test	N	Test statistic	Sig.
*Antisocial behaviour*			
Video games	2492	18.883	0.000**
Girls	1169	10.367	0.016*
Boys	1284	2.154	0.541
Online games	2476	29.516	0.000**
Girls	1160	9.833	0.020*
Boys	1277	0.721	0.868
Internet and social media	2475		
Girls	1162	94.207	0.000**
Boys	1274	84.764	0.000**
Mobile phones	2487	151.716	0.000**
Girls	1167	104.547	0.000**
Boys	1281	114.250	0.000**

_Significance is indicated by (*) for *p* < 0.05 and (**) for *p* < 0.01_

With regard to the differences observed between boys and girls, all the tests carried out (Table 2) rejected H0 and found significant differences in antisocial behaviour in relation to the use of video games, online games, internet, social media and mobile phones among girls. In the case of boys, significant differences were only observed in the use of the internet, social media and mobile phones.

**Fig. 1:**
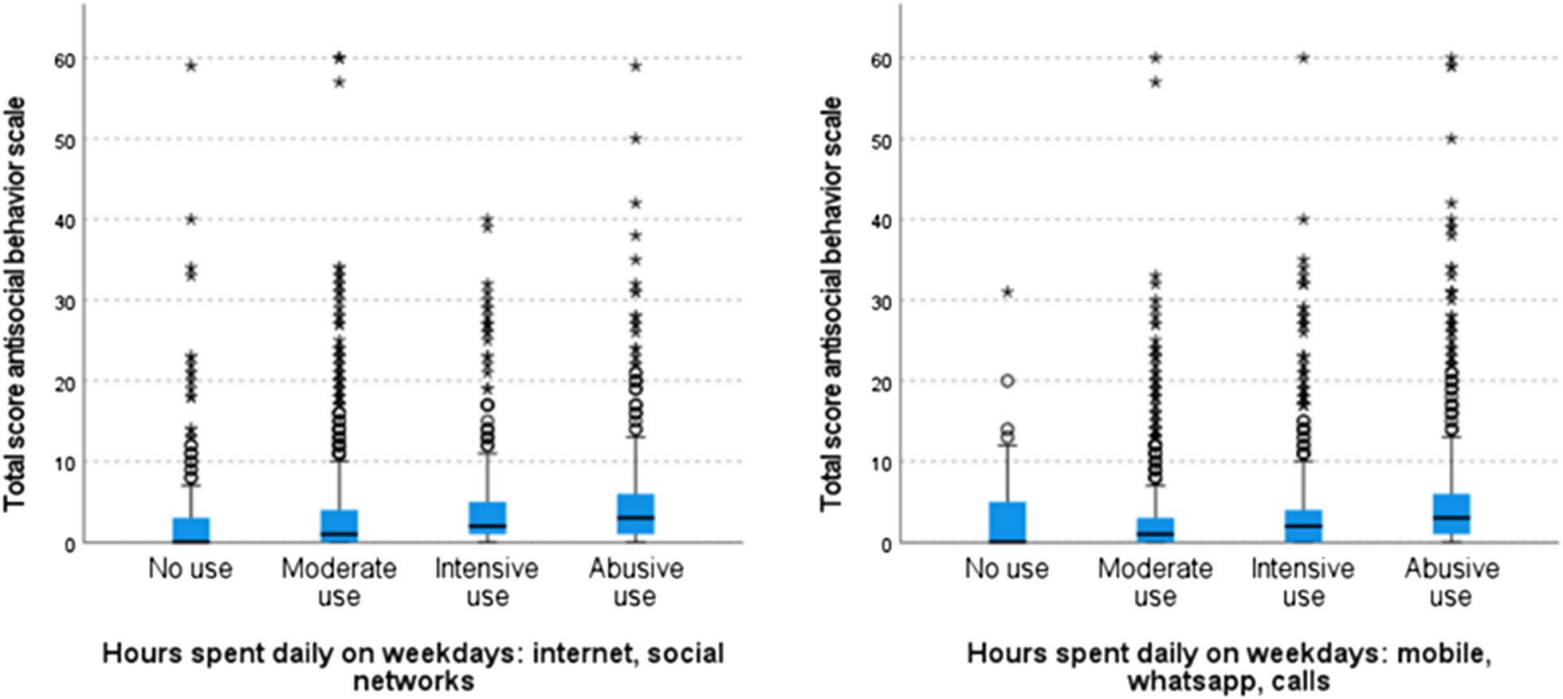
Box plot showing distribution of subsamples for antisocial behaviour (total sample)

These differences were primarily found in individuals making intensive use (2–4 h per day) or abusive use (> 4 h per day) of the different platforms (Table 3), who displayed higher levels of antisocial behaviour than others (Fig. 1).

**Table 3:** Significance values adjusted by Bonferroni correction of pairwise comparisons (total sample)

Sample 1–Sample 2	Video games	Online games	Internet and social media	Mobile phones
No use–moderate use	0.372	0.003**	0.000**	1.000
No use–intensive use	0.003**	0.000**	0.000**	0.532
No use–abusive use	0.012*	0.039*	0.000**	0.001**
Moderate use–intensive use	0.111	0.150	0.000**	0.000**
Moderate use–abusive use	0.161	1.000	0.000**	0.000**
Intensive use–abusive use	1.000	1.000	0.033*	0.000**

_Significance is indicated by (*) for *p* < 0.05 and (**) for *p* < 0.01_

With regard to bullying, all the tests carried out (see Table 4) rejected H_0_ and found significant differences in bullying in relation to the use of video games, online games, internet, social media and mobile phones.

**Table 4:** Contrasts for bullying hypothesis

Kruskal–Wallis test	N	Test statistic	Sig.
*Bullying*			
Video games	2626	51.733	0.000**
Girls	1231	7.218	0.065
Boys	1354	12.433	0.006*
Online games	2604	49.136	0.000**
Girls	1220	9.446	0.024*
Boys	1343	9.098	0.028*
Internet and social media	2606	23.888	0.000**
Girls	1224	31.771	0.000**
Boys	1341	20.223	0.000**
Mobile phones	2627	26.527	0.000**
Girls	1233	32.723	0.000**
Boys	1353	18.495	0.000**

_Significance is indicated by (*) for *p* < 0.05 and (**) for *p* < 0.01_

With regard to the differences observed between boys and girls, all the tests carried out (Table 4) rejected H_0_ and found significant differences in bullying in relation to the use of online games, internet, social media and mobile phones among girls. In the case of boys, significant differences were found in all the variables analysed.

**Fig. 2:**
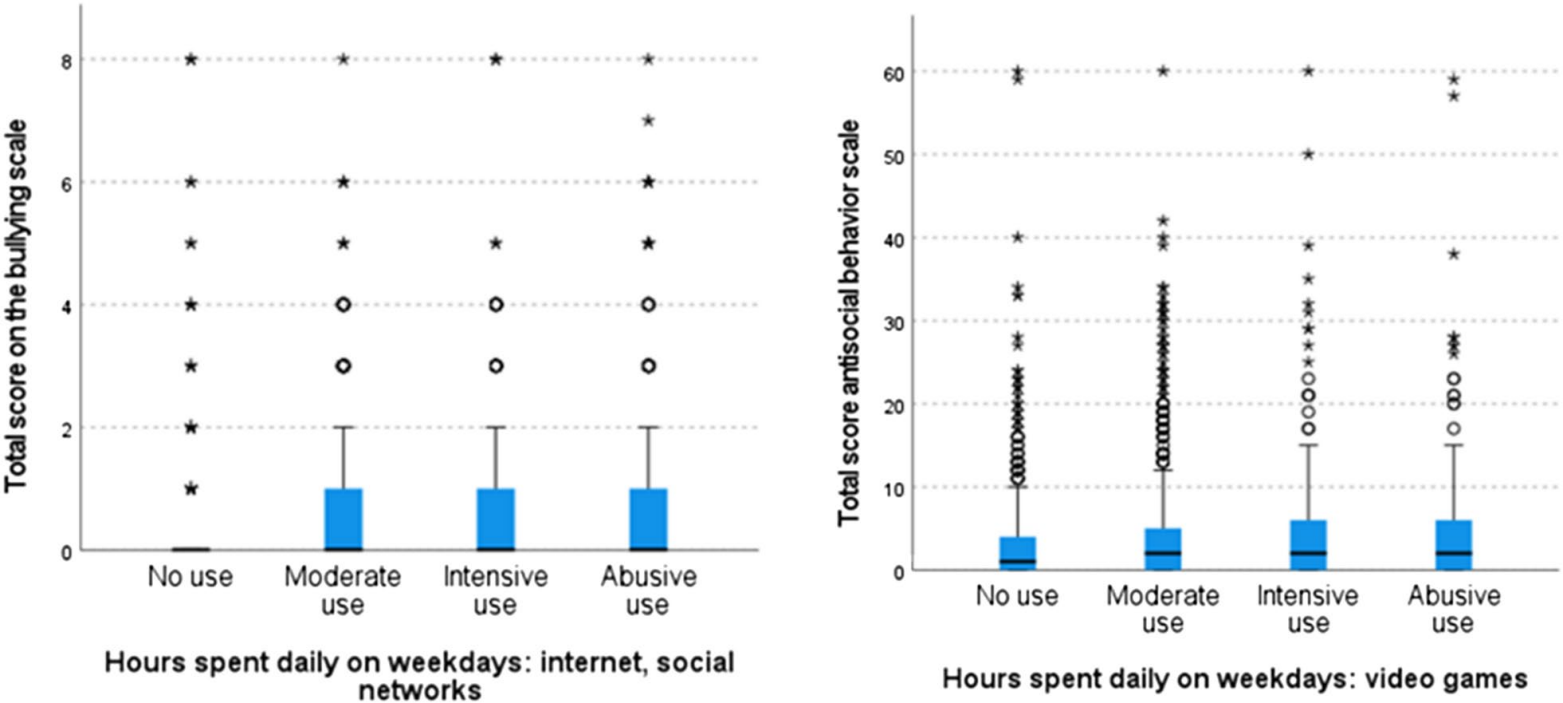
Box plot showing distribution of subsamples for bullying (total sample)

These differences were primarily observed (Table 5) between individuals who did not use these devices and those who did, with those engaging in intensive use (2–4 h per day) and abusive use (> 4 h per day) displaying significantly higher levels of bullying than the others (Fig. 2).

**Table 5:** Significance values adjusted by Bonferroni correction of pairwise comparisons (total sample)

Sample 1–Sample 2	Video games	Online games	Internet and social media	Mobile phones
No use–moderate use	0.000*	0.000**	1.000	0.182
No use–intensive use	0.000**	0.000**	0.670	0.440
No use–abusive use	0.000**	0.008**	0.000**	1.000
Moderate use–intensive use	0.096	0.004**	1.000	1.000
Moderate use–abusive use	0.485	0.969	0.000**	0.000**
Intensive use–abusive use	1.000	1.000	0.006**	0.003**

_Significance is indicated by (*) for *p* < 0.05 and (**) for *p* < 0.01_

The correlations between the variables were then analysed using Pearson’s chi-squared test, the Matthews correlation coefficient and the contingency coefficient. To do this, the variables related to antisocial behaviour and to bullying were categorised.

With regard to bullying, all tests performed rejected H0 and found significant associations between bullying and the use of video games, online games, the Internet, social media, and cell phones. As shown in Table 6, the Phi statistic is 0.143–0.195 out of a maximum possible value of 1. This represents a small effect size. This value is highly significant (*p* < 0.001), indicating that a value of the test statistic that is this large is unlikely to have occurred by chance, and therefore the strength of the relationship is significant. These results confirm what the chi-square test already told us, but also give us an idea of the size of the effect [65].

**Table 6:** Significant results in the correlation analysis between bullying and the use of information and communication technologies

	Video games	Online games	Internet and social media	Mobile phones
*Bullying*				
N	2626	2604	2606	2627
*Total score for scale*				
*X*2	86.257	98.687	56.591	53.842
Sig.	0.000**	0.000**	0.000**	0.000**
C	0.178	0.191	0.146	0.142
Ø	0.181	0.195	0.147	0.143
Sig.	0.000**	0.000**	0.000**	0.000**

_Significance is indicated by (**) for *p* < 0.01_

With regard to antisocial behaviour, all the tests carried out (see Table 7) rejected H0 and found significant associations between antisocial behaviour and the use of video games, online games, internet, social media and mobile phones.

**Table 7:** Significant results in the correlation analysis between antisocial behaviour and the use of information and communication technologies

	Video games	Online games	Internet and social media	Mobile phones
*Antisocial behaviour*				
N	2492	2476	2475	2487
*Total score for scale*				
*X*2	231.166	285.256	275.861	280.424
Sig.	0.000**	0.000**	0.000**	0.000**
C	0.291	0.321	0.317	0.318
Ø	0.305	0.339	0.334	0.336
Sig.	0.000**	0.000**	0.000**	0.000**

_Significance is indicated by (**) for *p* < 0.01_

As shown in Table 7, the Phi statistic is 0.305–0.339 out of a maximum possible value of 1. This represents a medium effect size [65].

## Discussion and conclusion

Considering the variables selected to approach this reality, and the limitations of the questionnaires used, already detailed in the methodological section, the results of this study show that the antisocial behaviour and bullying variables are associated with the different categories of use of information and communication technology. More prevalent use of these technologies (abusive use) is correlated with higher scores on the scales measuring antisocial behaviour and bullying. However, this in no way implies that these associations are causal; rather, they are concurrent in situations where adolescents engage in antisocial behaviour or bullying. These results are related to explanations such as those of Domínguez and Portela [50] about violence in social networks being increasingly widespread among young people due to the importance of features such as anonymity [54].

Based on the findings of this exploratory study, gender differences appear to be irrelevant in this correlation as it is similar in both boys and girls. According to the scientific literature [50, 52, 53], antisocial behaviour and bullying are more prevalent and intense among boys than girls. However, our analysis suggests that the direct correlation between these behaviours and the use of information and communication technology operates in a similar manner in both genders. This does not mean that we reject the idea posited by Muñiz et al. [55] with regard to differences in the use of ICT by gender, but this would require a different kind of analysis to explore fully.

Numerous studies have shown that antisocial behaviour and bullying or cyberbullying are influenced by a range of factors, including abusive use of ICT [66] and the normalisation of violence by video games or the internet, as indicated by Cowie [56]. The level of violence observed in the online world reinforces aggressive, violent behaviour in the real world [67].

Similarly, adolescents’ involvement in these types of violent behaviour is determined by other risk factors, including loneliness, depression, low self-esteem and poor academic performance, among others [27, 28]. These factors encourage further involvement in unhealthy behaviours, as Schenk and Fremouw [57] explain. In this sense, a consideration for future studies would be to employ statistical tools (i.e., polychoric correlations, item factor analysis and structural equation modeling) specifically developed to analyze latent variables measured with ordinal categorical items as a way to deepen the approach from simply testing null hypotheses to testing alternative theoretical models [68].

These data must be taken into consideration by the educational community, which has been shown to have a protective influence [69–72] and by school guidance departments in particular, which are designed specifically to tackle these kinds of situations. Each autonomous community has a different protocol for dealing with bullying and cyberbullying and there is evidence of some successful programmes such as the internationally known anti-bullying programme KIVA. The existing literature and the results of this study point to the need to continue to deepen preventive measures to educate students in peaceful coexistence, to provide a modern and inclusive education and to allow each student to fulfil themselves, rejecting violence and adopting creative and participatory ways of dealing with conflicts.

With regard to the use of ICT specifically, educational interventions should acknowledge that these technologies are here to stay and that they form part of adolescents’ daily lives. ICT should be viewed as a tool to satisfy the need for autonomy, new sensations and affective bonds among adolescents [19, 20] a perspective that remains unfamiliar to many professionals. Technological tools offer adolescents emotional support and a sense of belonging [21], creating a new venue where they can socialise with their peers as they would once have done in their neighbourhoods or streets [20, 24].

In addition, it is important for schools to recruit staff to roles that focus on addressing matters relating to peaceful coexistence in a holistic manner. Roles such as social educator will allow programs to prevent bullying and create harmonious educational environments to be planned, coordinated, implemented and evaluated. The General Council of Associations of Social Educators [8] has listed a series of proposals for social education: training students as (cyber)mediators, training and guidance for teachers on dealing with bullying and cyberbullying, measures to increase educational success, conflict resolution through dialogue, preventive socialization to avoid gender-based violence, emotional education programs, liaising with community leaders, etc. Formal education is vitally important, but it is also crucial that action is taken in more informal spaces to build, rebuild and add the necessary elements to ensure that the educational community becomes a genuine driver of social change.

Based on the contributions made in this article, the aim of which was to find out, through an exploratory analysis, the relationship between the abusive use of the internet and social networks and involvement in violent behaviour, we consider it interesting to propose future research studies that analyse the relationship between adolescent and youth risk behaviours, rather than the development of specific behaviours in an isolated and individual manner. Likewise, we consider it relevant to continue delving into more current and novel risk behaviours such as those related to the use and abuse of information and communication technologies, and the role that the gender of adolescents and young people plays in these processes, without forgetting the impact that the peer group has in this vital stage [1, 20, 25].

Information and communication technologies are here to stay, and can be potential educational tools, but we must recognise that it is necessary to investigate and understand the impact they have on the development of addictive behaviour and involvement in violent behaviour among the adolescent-youth population. For this reason, it is proposed to continue with research that on the one hand brings us closer to the current situation of young people, and on the other hand, that can make innovative and close approach proposals, which will have an impact on the target population, to try to minimise the negative impact, without forgetting the need to offer education and training to the adult population (family, school and community), so that we can, in a joint and coordinated way, prevent the associated risks, and delve into the potentialities [1, 36].

## Data Availability

The datasets generated and/or analyzed during the present study are not publicly available because they belong to the public administration (Basque Government), but are available from the corresponding author upon reasonable request.

## Footnotes

1 In relation to the procedure, this study has been developed respecting current regulations and principles of ethics related to the protection and avoidance of risks to participants and respect for autonomy
